# Allelic Variant of Armadillo Repeat Containing Protein 5 (ARMC5) in Myelolipoma Mimicking Pheochromocytoma: A Case Report

**DOI:** 10.7759/cureus.34454

**Published:** 2023-01-31

**Authors:** Nejat Naser, Victor Nava, Shikha Khosla, Eric Nylen

**Affiliations:** 1 Department of Endocrinology, George Washington University School of Medicine, Washington, USA; 2 Department of Endocrinology, Veterans Affairs Medical Center, Washington, USA; 3 Department of Pathology, The George Washington University, Washington, USA; 4 Department of Pathology, Veterans Affairs Medical Center, Washington, USA; 5 Department of Endocrinology Diabetes and Metabolism, George Washington University, Washington, USA; 6 Department of Endocrinology, Diabetes and Metabolism, Veterans Affairs Medical Center, Washington, USA; 7 Department of Endocrinology and Diabetes, George Washington University, Washington, USA

**Keywords:** case report, adrenal nodule, pheochromocytoma, myelolipoma, armadillo repeat containing protein 5 (armc5) gene

## Abstract

Adrenal myelolipomas are benign adrenocortical tumors composed of adipose tissue mixed with hematopoietic precursor cells. An association of myelolipoma with adrenal cortical adenoma is rare and the pathogenesis of these tumors remains unclear. Here we present a case of an incidentally discovered adrenal tumor with radiologic characteristics of a myelolipoma who underwent adrenalectomy due to biochemical suspicion for pheochromocytoma. The final pathology, however, revealed a myelolipoma with a co-existing adrenal cortical adenoma without evidence of pheochromocytoma. Genetic analysis revealed the presence of a hitherto unreported heterozygous variant, c.329C>A (p.Ala110Asp), of the armadillo repeat-containing protein 5 (ARMC5) gene which when inactivated is commonly associated with bilateral adrenal nodularity.

## Introduction

Myelolipomas account for approximately 4%-7% of all adrenal tumors. They are usually unilateral and are generally considered non-secretory. The pathogenesis of these tumors remains unclear but current suggested etiologies include: 1) metaplasia of reticuloendothelial cells in the adrenal glands due to stress, infection, or trauma, 2) embolism of bone marrow cells to the adrenal gland, and/or 3) chronic ACTH stimulation of adrenal cells. Rarely these tumors can co-exist with functional or non-functional adrenocortical adenomas and have been associated with endocrine disorders including Cushing syndrome, congenital adrenal hyperplasia (CAH), Conn’s syndrome, and pheochromocytoma [1-4].

The armadillo repeat containing 5 (ARMC5) gene is a tumor suppressor gene, located on chromosome 16 in humans, and codes for a 771-aminoacid protein with an armadillo repeat domain believed to be involved in adrenal hormone homeostasis and adrenal cell apoptosis. Inactivating mutations of ARMC5 are implicated in the pathogenesis of primary bilateral macronodular hyperplasia (PBMH). Non-pathogenic allelic variants of ARMC5 have also been discovered in unilateral functional and non-functional adrenal nodules. However, ARMC5 association with myelolipomas or mixed tumors has not been reported [5,6].

## Case presentation

**Figure 1:**
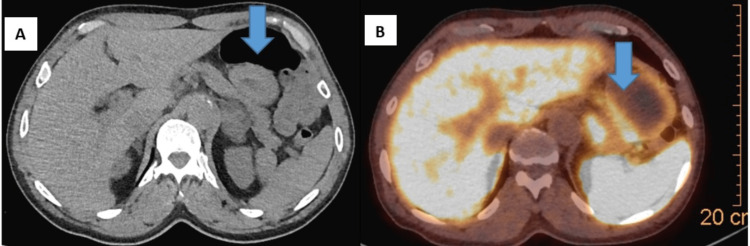
(A) CT scan of the abdomen and pelvis with and without contrast. A 4.1 x 3.1 cm left adrenal mass (arrow) with focus of macroscopic fat posteriorly highly supportive of adrenal myolipoma. (B) On GA-68 DOTATATE in the medial inferior aspect, there is relatively focal, moderate to intense radiotracer activity noted (Krenning score 3).

A 61-year-old man with a medical history of hepatitis C infection, liver cirrhosis, hypertension, and post-traumatic stress disorder was referred to endocrinology for evaluation of an adrenal nodule. During a routine CT scan of the abdomen for evaluation of painless jaundice, the patient was noted to incidentally have a 3.8 cm left adrenal nodule (the right adrenal gland was normal). A follow-up adrenal CT scan noted a 4.1 x 3.1 cm left adrenal mass, HU <10, with evidence of macroscopic fat and punctate areas of calcification consistent with adrenal myelolipoma (Figures 1A, 1B).

Clinically, the patient’s blood pressure was well controlled on spironolactone and furosemide and he did not have signs concerning hypercortisolism or pheochromocytoma. The initial biochemical workup (Table 1) revealed abnormal dexamethasone suppression test with cortisol of 2.2 µg/dL (normal: <1.8) despite an adequate serum dexamethasone level of 660 ng/dL. A 24-h urinary free cortisol was minimally elevated 53.7 mcg/24h (normal: 4-50mcg/24h). Plasma ACTH was normal. Plasma metanephrine was twice the upper limit of normal at 201 pg/mL (normal: <57 pg/mL) but normetanephrine was normal. Subsequently, repeat biochemical testing being off anti-depressants for over eight months showed that plasma metanephrine remained elevated at 128 pg/mL (normal: <57 pg/mL) with normetanephrine again being normal. Plasma epinephrine level was also elevated at 172 pg/mL (normal 0-50 pg/mL). A repeat dexamethasone test revealed a serum cortisol of 2.2 µg/dL (normal: <1.8) with an adequate dexamethasone level of 478 ng/dL. However, repeat 24-hour urinary cortisol was normal (done twice) at a level of 12.3 mg/24 h and 24.9 mcg/24 h (normal: 4-50 mcg/24 h). In addition, late-night salivary cortisol (also done twice) was normal as well with levels below 0.03 mcg/dL (normal: <0.09 mcg/dL).

**Table 1:** Pertinent laboratory data before and after surgery

Test	Pre-operative Level	Post-operative Level	Reference range
ACTH	11 pg/mL	35 pg/mL	6-50 pg/mL
Dexamethasone suppression test 1: Cortisol level with plasma Dexamethasone level of 660ng/dL	2.2	Not done	<1.8 µg/dL
Dexamethasone suppression test 2: Cortisol level with plasma Dexamethasone level of 478ng/dL	2.2	Not done	<1.8 µg/dL
Salivary cortisol x 2	<0.03 mcg/dL	Not done	<0.09 mcg/dL
24-hour urinary free cortisol 1	53.7 mcg/24 hr	Not done	4-50 mcg/24 hr
24- hour urinary free cortisol 2	12.3 mcg/24 hr	Not done	4-50 mcg/24 hr
24- hour urinary free cortisol 3	24.9 mcg/24 hr	Not done	4-50 mcg/24 hr
Plasma Metanephrines 1	201 pg/mL	56 pg/mL	<57 pg/mL
Plasma Metanephrines 2	128 pg/mL (off anti-depressants)	44 pg/mL	<57 pg/mL
Plasma Normetanephrines 1	113 pg/mL	71 pg/mL	<148 pg/mL
Plasma Normetanephrines 2	97 pg/mL	67 pg/mL	<148 pg/mL
Plasma epinephrine	172 pg/mL	127 pg/mL	0-50 pg/mL

**Figure 2:**
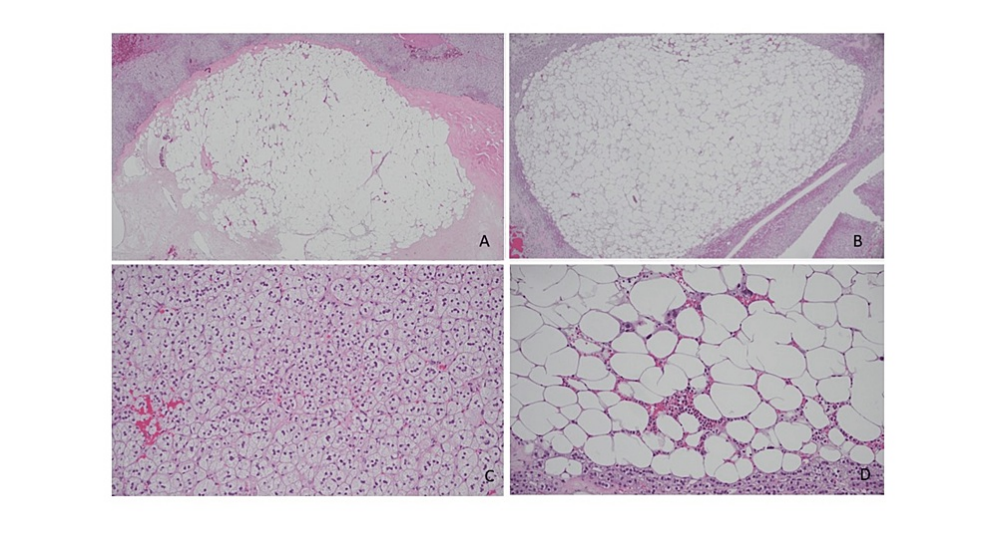
(A) Myelolipoma with areas fibrosis (Hematoxylin and eosin stain, 20x); (B) mature adipose tissue rich-area of myelolipoma (Hematoxylin and eosin stain, 40x); (C) hematopoietic rich-area of myelolipoma (Hematoxylin and eosin stain, 200x); (D) adrenal cortical adenoma resembling zona fasciculata (Hematoxylin and eosin stain, 200x).

Given the persistent elevation in plasma metanephrines, the patient underwent Ga-68 DOTATE scan which showed a 4.1 x 3.6 cm left adrenal mass with heterogeneous uptake showing moderate to intense radiotracer activity in the medial inferior, and a mild to moderate activity the peripheral aspect of the mass. These findings raised concern for neuroendocrine component of the mass. Follow up MRI of the abdomen with adrenal protocol revealed significant heterogeneity in the 4.1 x 3.6 cm left adrenal mass. Given the tumor size, elevated plasma metanephrines, and radiologic characteristics, patient underwent left adrenalectomy after appropriate pre-op optimization including alpha blockade. The final pathology revealed a 6 x 4 x 4 cm mixed myelolipoma and adrenocortical adenoma (Figures 2A-2D) without evidence of pheochromocytoma on complete submission of tissue.

Interestingly, post-operative plasma metanephrine normalized to 56 pg/mL (normal: <57 pg/mL), normetanephrine of 71 (normal: <148 pg/mL) and Total metanephrines of 127 pg/mL (normal: <205 pg/mL), and he was taken off alpha blockade. Genetic sequencing (Fulgent Laboratories) revealed a novel heterozygous variant, c.329C>A (p.Ala110Asp) of unknown clinical significance consisting of a single amino acid substitution (missense) of ALA to ASP of codon 10 in exon 1 of the ARMC5 gene.

## Discussion

The current case illustrates a mixed myelolipoma-cortical adenoma mimicking biochemical features of pheochromocytoma leading to adrenalectomy. Interestingly, gene sequencing of the ARMC5 gene revealed a hitherto unreported genetic variant.

Myelolipomas are typically asymptomatic, remain stable in size over time, and typically do not require surgical intervention [3]. Moreover, hormonal evaluation for myelolipoma is not recommended as they do not contain functional adrenal tissue and are generally considered non-secretory [7]. However, hormone secretion is expected when coexisting adrenocortical adenoma is present. In one comprehensive review of myelolipoma with 440 patients, CAH and adrenal hypersecretory disorders (cortisol and aldosterone) were found in 10% and 7.2% of cases respectively. In addition, two patients harbored pheochromocytomas, and androgen secretion was reported in four cases. Furthermore, complaints and symptoms of hypercortisolism, hyperaldosteronism, and hyperandrogenism were reportedly resolved after myelolipoma resection, supporting the collision of myelolipomas with other functional adrenal lesions [2]. Accordingly, in our case, metanephrine completely normalized following adrenalectomy.

The ARMC5 protein is a tumor suppressor that is located on chromosome 16p11.2 and has been implicated in non-ACTH dependent Cushing syndrome due to PBMH, where inactivating mutations lead to dedifferentiation of adrenocortical cells and growth of bilateral masses. Patients who harbor ARMC5 mutations have severe disease, including large adrenal glands, numerous nodules, higher cortisol levels, and more severe hypertension. In addition, ARMC5 mutations have been associated with primary aldosteronism and may also play a role in the development of meningiomas [4]. In a study that reviewed 59 patients with unilateral adrenal incidentalomas almost 70% of patients were found to have a non-pathogenic allelic variant of ARMC5 as well [5]. However, ARMC5 variants have not been reported in myelolipoma or mixed tumors. In the case of myelolipoma-associated CAH, it has been suggested that hyperplasia was related to ACTH stimulation [1]. Alternatively, adrenal growth may have been related to the ARMC5 mutation.

In view that PBMH is recognized to be associated with tumor syndromes such as familial adenomatous polyposis coli, multiple endocrine neoplasia type 1, and fumarate hydratase germline mutations, genetic counseling, and familial evaluation were recommended for our patient [8].

## Conclusions

Myelolipomas are incidentally discovered as adrenal incidentalomas that are almost always unilateral. These tumors can be associated with other adrenal neoplasms and in rare circumstances, can be secretory, suggesting composite lesions. Hormonal evaluation is not indicated for myelolipomas according to current guidelines, but depending on the clinical picture hormonal evaluation needs to be considered. The etiology of myelolipomas remains debatable and further studies are warranted to evaluate the association of ARMC5 mutations with adrenal myelolipomas or mixed tumors. In view that PBMH is recognized to be associated with tumor syndromes such as familial adenomatous polyposis coli, multiple endocrine neoplasia type 1, etc., genetic counseling and familial evaluation should be offered to all patients.
